# Molecular mechanism of hyperactive tooth root formation in oculo-facio-cardio-dental syndrome

**DOI:** 10.3389/fphys.2022.946282

**Published:** 2022-07-25

**Authors:** Kyaw Min Soe, Takuya Ogawa, Keiji Moriyama

**Affiliations:** Department of Maxillofacial Orthognathics, Graduate School of Medical and Dental Sciences, Tokyo Medical and Dental University, Tokyo, Japan

**Keywords:** BCL6, BCOR, OFCD syndrome, radiculomegaly, ZFPM2

## Abstract

Mutations in the B-cell lymphoma 6 (BCL6) interacting corepressor (BCOR) cause oculo-facio-cardio-dental (OFCD) syndrome, a rare X-linked dominant condition that includes dental radiculomegaly among other characteristics. BCOR regulates downstream genes *via* BCL6 as a transcriptional corepressor. However, the molecular mechanism underlying the occurrence of radiculomegaly is still unknown. Thus, this study was aimed at identifying BCOR-regulated genetic pathways in radiculomegaly. The microarray profile of affected tissues revealed that the gene-specific transcriptional factors group, wherein nucleus factor 1B, distal-less homeobox 5, and zinc finger protein multitype 2 (ZFPM2) were the most upregulated, was significantly expressed in periodontal ligament (PDL) cells of the diseased patient with a frameshift mutation (c.3668delC) in BCOR. Wild-type BCOR overexpression in human periodontal ligament fibroblasts cells significantly hampered cellular proliferation and ZFPM2 mRNA downregulation. Promoter binding assays showed that wild-type BCOR was recruited in the BCL6 binding of the ZFPM2 promoter region after immunoprecipitation, while mutant BCOR, which was the same genotype as of our patient, failed to recruit these promoter regions. Knockdown of ZFPM2 expression in mutant PDL cells significantly reduced cellular proliferation as well as mRNA expression of alkaline phosphatase, an important marker of odontoblasts and cementoblasts. Collectively, our findings suggest that BCOR mutation-induced ZFPM2 regulation *via* BCL6 possibly contributes to hyperactive root formation in OFCD syndrome. Clinical data from patients with rare genetic diseases may aid in furthering the understanding of the mechanism controlling the final root length.

## Introduction

Oculo-facio-cardio-dental (OFCD) syndrome (OMIM 300166) is a rare X-linked genetic disorder characterized by microphthalmia, congenital cataracts, facial dysmorphic features, congenital heart defects, and dental anomalies; the latter includes teeth with excessively long roots, or radiculomegaly (Gorlin et al., 1996; Hedera and Gorski 2003; Horn et al., 2005; Oberoi et al., 2005). Radiculomegaly in particular has long been considered a key symptom in the diagnosis of OFCD syndrome (Larhant et al., 2014; Zhu et al., 2015). OFCD syndrome is caused by mutations in the B-cell lymphoma 6 (BCL6) interacting corepressor (BCOR) gene, which is located in the Xp11.4 region and encodes the BCL6 corepressor (Zhu et al., 2015). BCOR is a transcriptional corepressor that interacts with the POZ/BTB domain of transcriptional BCL6 in the nucleus, which is required for cell proliferation and apoptosis. BCOR also acts as a key transcriptional regulator in early embryogenesis (Huynh et al., 2000).

Almost all the identified OFCD syndrome mutations include nonsense, frameshifting insertion/deletion/duplications, splicing mutations, and substantial deletions (Surapornsawasd et al., 2014; Ragge et al., 2019). Frameshift or nonsense mutations result in the production of premature termination codons (PTCs) that cause inherited genetic diseases. Most nonsense transcripts are destroyed by nonsense-mediated mRNA decay (NMD), a surveillance system in eukaryotes that protects the organism from deleterious dominant-negative or gain-of-function effects of truncated proteins. Depending on the position of the PTC in the transcript and the characteristics of the shortened protein, NMD may have a beneficial, neutral, or detrimental effect (Frischmeyer and Dietz, 1999). According to our previous study, mutations leading to PTC-induced NMD in the periodontal ligament (PDL) cells caused unstable mutant transcripts and increased cell proliferation, which could contribute to hyperactive root formation (Surapornsawasd et al., 2014).

Compared with that of crown formation, the molecular mechanism of root formation is poorly understood. The apical papilla, which originates from the ectomesenchyme, is responsible for the complete elongation and maturation of the dental root. During root growth, the apical portion of the enamel organ elongates, forming a bilayer epithelial structure known as Hertwig’s epithelial root sheath (HERS). The interaction of stem cells from the apical papilla close to HERS is detected at the apical region of root development, which regulates apical root morphogenesis and determines root number, length, and dentin production. HERS elongation is mediated by several signaling mechanisms, and the PDL tissues contain epithelial cell rests of Malassez (ERM), which are formed from HERS fragments during root formation (Zeichner-David et al., 2003; Tsunematsu et al., 2016). Changes in the activation of these critical signaling pathways can have a significant impact on tooth growth, including shortened or absent roots (Kollar and Baird, 1970).

Notably, all other anomalies in OFCD syndrome, aside from radiculomegaly, appear to be “deficiency” defects, such as cardiac septal defects, hypodontia, and cleft palate. Continuous root growth appears to have the opposite effect (Gorlin et al., 1996; Hedera & Gorski, 2003; Horn et al., 2005; Oberoi et al., 2005; Larhant et al., 2014; Zhu et al., 2015). Human organ size is controlled by certain mechanisms, including the closure of the anterior and posterior neuropores (Nikolopoulou et al., 2017). The teeth of patients with OFCD syndrome appear to be deficient in this regulatory system, resulting in continued root growth. In the case of OFCD syndrome, disruption of the control of root length may be attributed to the activation of downstream molecules by the dysfunction of BCOR as a corepressor. We expected that a BCOR corepressor mutation would result in radiculomegaly due to a failure to regulate its downstream genes during normal dental root formation. To resolve this critical issue, we performed gene expression profiling using an OFCD patient with a frameshift mutation (c.3668delC) in the BCOR gene, as well as in a healthy subject, to identify BCOR target genes that might be related to downstream target genes. In gene-specific transcriptional regulatory protein groups, zinc finger protein multitype 2 (ZFPM2) was significantly upregulated in OFCD patients. Our findings contribute to the current knowledge on the abnormal gene upregulation that occurs in response to the decline in the BCOR corepressor in patients with OFCD syndrome; we also show that ZFPM2 is a plausible downstream gene involved in radiculomegaly.

## Materials and methods

### Patient selection and cell culture

An 18-year-old patient with a c.3668delC frameshift mutation in the BCOR gene and a 16-year-old healthy female subject were included in this study. Patient details have been described previously (Surapornsawasd et al., 2014; Myat et al., 2018). The first premolars of both the patient and the healthy subject were extracted for orthodontic reasons. The PDL surrounding the apical 1/3 of the root tissues was harvested using an aseptic technique and then cultured in α-MEM (Wako, Osaka, Japan) supplemented with 10% fetal bovine serum (Gibco, Life Technologies, Paisley, United Kingdom) and penicillin-streptomycin (Gibco) as previously described (Surapornsawasd et al., 2014; Myat et al., 2018). The experiments involving human subjects described herein were approved by the Ethical Review Committee of Tokyo Medical and Dental University (No. D2014-002). Both participants provided written informed consent to participate in the study.

### Microarray analysis and gene ontology and pathway analysis

Total RNA was extracted from the cultured PDL cells of the OFCD patient and healthy subject using the QiagenRNeasy^®^ Mini Kit (Qiagen, Hilden, Germany) according to the manufacturer’s instructions. RNAs extracted from OFCD PDL cells and healthy PDL cells were sequenced with human whole-genome microarrays using 3D-Gene™ Human Oligo Chip 25 k (Toray Group Kamakura Techno-Science, Inc.). Entrez gene IDs for upregulated gene lists were used as the initial dataset for enrichment analysis. The protein analysis through evolutionary relationships (PANTHER) classification system was used to classify differentially expressed genes to facilitate data analysis and intuitive visualizations for gene ontology (GO) analysis output. GO and pathway enrichment analyses were performed to predict the functions of differentially expressed genes in OFCD patients and healthy subjects. All microarray data were deposited in a MIAME-compliant database, the National Center for Biotechnology Information Gene Expression Omnibus (GSE19870), as described on the Microarray and Gene Expression Data Society website.

### Plasmid construction and transfection

A wild-type (WT) BCOR plasmid, mutated (Mut) BCOR plasmid with a c.3668delC frameshift mutation in the BCOR gene, and an empty plasmid described in our previous study were used (Surapornsawasd et al., 2015). Primary human PDL fibroblasts, HPdLF (Lonza, Walkersville, MD, United States), and COS-7 cells were grown and cultured in SCGM BulletKit™ medium (Lonza) and α-MEM (Wako), respectively; they were kept in a humidified 5% CO_2_ atmosphere at 37°C. Overexpression of each plasmid was performed using Lipofectamine P3000 reagent (Invitrogen, Van Allen Way Carlsbad, CA, United States) according to the manufacturer’s instructions. After 24 h, immunofluorescence and cell proliferation assays were performed. All experiments using the expression plasmids were approved by the Recombinant Experiment Committee of our institution (G2020-021A).

### Quantitative reverse transcription polymerase chain reaction

Quantitative reverse transcription polymerase chain reaction (qRT-PCR) was performed using the Power SYBR Green PCR master mix (Applied Biosystems, Foster City, CA, United States) according to the manufacturer’s instructions on an ABI 7,000 system (Applied Biosystems). At least three replicates were performed for each sample, and relative gene expression was calculated using the 2^−ΔΔCt^ method (Livak and Schmittgen, 2001). The sequences of the primers used are listed in the Supplementary File.

### Immunofluorescence assay

The WT BCOR plasmid was transfected into HPdLF cells cultured on Thermo Scientific Lab-Tek II chamber slides (Thermo Fisher Scientific Inc., Rochester, NY, United States) at a 70% confluent cell density. The empty plasmid was used as a control. Cells were washed twice with phosphate-buffered saline (PBS) and fixed by precooling methanol at −10°C for 5 min before blocking for 30 min with 10% goat serum in PBST. The cells were then incubated with the primary mouse monoclonal anti-FLAG M2 antibody (1:1000; Sigma–Aldrich, St. Louis, MO, United States) for 45 min, followed by incubation with the secondary goat anti-mouse IgG-FITC antibody (1:1000; Santa Cruz Biotechnology, Santa Cruz, CA, United States) for 45 min. Glass slides were mounted with Prolong Gold antifade reagent with DAPI (Life Technologies, Carlsbad, CA, United States), and cells were observed using a Leica AF6000 fluorescence microscope (Leica Microsystems, Wetzlar, Germany). The Flag-tagged BCOR and Mut BCOR with a frameshift mutation in c.3668delC protein were successfully detected with a monoclonal anti-FLAG M2 antibody using immunofluorescence assay and western blot analysis as described by us previously (Surapornsawasd et al., 2015). Experiments were performed in triplicate to obtain reliable results.

### MTT assay

Cells were cultured at a concentration of 1,000 cells per 100 μl in each well of 96-well plates. Eight wells were replicated for each group, and wells containing culture medium were used as the negative control. Cells were incubated at 37°C for 0, 12, 24, 36, and 48 h in a humidified 5% CO_2_ atmosphere. Each well was incubated with 20 μl of 5 mg/ml MTT (Sigma-Aldrich) at 37°C for 3.5 h, and the solution was removed. Then, 150 μl of MTT solvent (4 mM HCl and 0.1% Nonidet P-40 in isopropanol) was added, and absorbance was measured at 590 nm with a reference value of 680 nm using a Bio-Rad model 680 microplate reader (Bio-Rad, Hercules, CA, United States). The cell proliferation rate was calculated by taking the average of the duplicate readings for each sample and subtracting the background absorbance of the culture medium, which provided the corrected absorbance. The absorbance determined was proportional to the number of cells. Experiments were performed in triplicate to obtain reliable results.

### Chromatin immunoprecipitation assay

The chromatin immunoprecipitation (ChIP) assay was performed using an Abcam High Sensitivity kit (#ab185913; Abcam) according to the manufacturer’s instructions. Using the Lipofectamine 3000 reagent, cells were transfected with WT BCOR, Mut BCOR, and empty plasmids. After 24 h, the cells were washed twice with PBS and incubated for 15 min at room temperature (20–25°C) with 1% formaldehyde, followed by another 5 min incubation with 125 mM glycine. Thereafter, the cells were washed twice with PBS. Mouse monoclonal anti-FLAG M2 (Sigma-Aldrich) or control IgG (#ab185913; Abcam) was used for immunoprecipitation. qRT-PCR was used to determine the relative enrichment of promoters using primers for the ZFPM2 promoter region containing BCL6 binding sites.

### RNA silencing

We knocked down BCL6 and ZFPM2 using Lipofectamine P3000 (Invitrogen) according to the manufacturer’s instructions. BCL6 siRNA (sc-29791; Santa Cruz Biotechnology), control siRNA (sc-37007; Santa Cruz Biotechnology), and ZFPM2 siRNA (sc-35399; Santa Cruz Biotechnology) were used in this study. qRT-PCR was performed to evaluate the effects of siRNA silencing.

### Statistical analysis

Data are presented as the mean ± standard deviation of three biological replicates. Three biological replicates were performed for mRNA extraction from the cell lines and cell proliferation. The normality data were initially analyzed using the Shapiro–Wilk normality test. Because the data were normally distributed, the significance of the average values was analyzed using a *t*-test or ANOVA (variable). A *p*-value of < 0.05 was considered significant. Statistical analyses were performed using Prism 9 (GraphPad Software, Inc., La Jolla, CA, United States).

## Results

### Microarray gene profiling and *in vitro* assay of HPdLF cells

**FIGURE 1:**
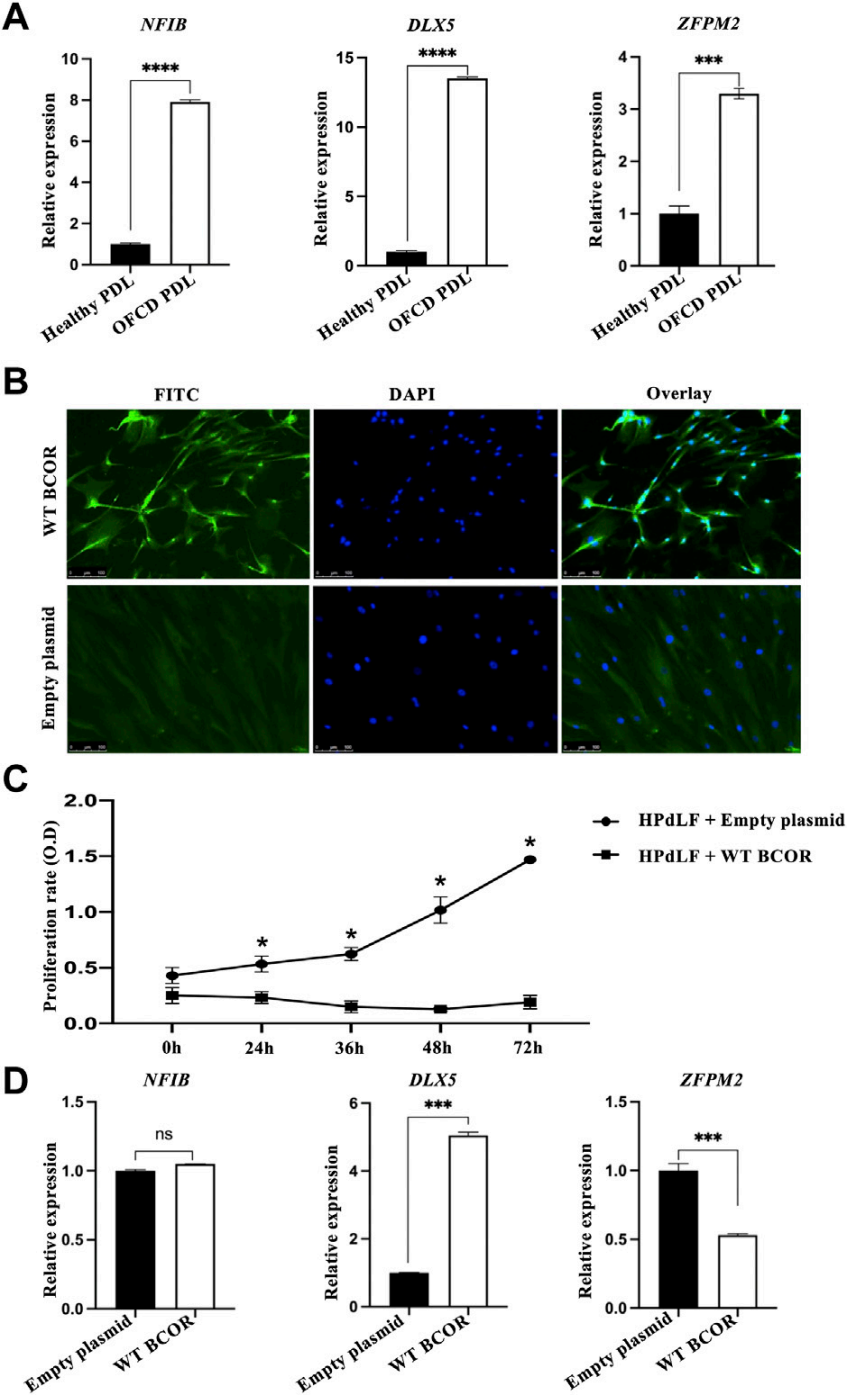
Microarray gene profiling and *in vitro* assay of HPdLF cells. **(A)** qRT-PCR validation of microarray analysis for upregulating genes with a fold change greater than 15. Expression levels of *ZFPM2*, *NFIB*, and *DLX5* relative to GAPDH expression were determined. Data are expressed as the means ± standard deviations; *t*-test, ****p < 0.0001, ***p < 0.001. **(B)** Overexpression of WT BCOR in HPdLF cells *in vitro.* HPdLF cells stably expressing WT BCOR were confirmed using an immunofluorescence assay. HPdLF cells were immunostained with anti-FLAG antibody and FITC-labeled secondary antibodies. Cell nuclei were counterstained with DAPI. An empty plasmid was used as a negative control. Samples were analyzed and photographed using an epifluorescence microscope. Scale bars represent 100 μm. **(C)** The MTT assay revealed that WT BCOR transfection suppressed HPdLF cell proliferation compared to transfection with empty plasmids. Points and bars indicate means and standard deviations, respectively, of the triplicate values. ANOVA, *p < 0.05. **(D)** qRT-PCR analysis of *NFIB*, *DLX5*, and *ZFPM2* expression after WT BCOR overexpression. Data are expressed as means ± standard deviations; *t*-test, ***p < 0.001; ns, not significant.

In a previous study, we showed that NMD observed in human PDL cells from patients with OFCD syndrome may be involved in hyperactive root formation (Surapornsawasd et al., 2014). Therefore, we investigated the gene expression profiles of PDL cells from patients with and without OFCD syndrome to identify the downstream components of the molecular mechanism by which BCOR regulates radiculomegaly in OFCD syndrome. In total, 1,249 genes were identified to be differentially expressed in Mut PDL cells compared with those in WT cells, with 874 genes upregulated (fold change > 2, *p* < 0.05) and 375 genes downregulated (fold change < 2, *p* < 0.05). The number of upregulated genes was significantly higher than that of downregulated genes in Mut PDL cells. Because BCOR is a transcriptional corepressor, we focused on upregulated genes to find its downstream molecules. The most significantly upregulated protein classes or genes in OFCD syndrome were extracellular matrix proteins, chromatin/chromatin-binding or regulatory binding proteins, cytoskeletal proteins, cell adhesion proteins, ion channels, and gene-specific transcriptional regulatory proteins (Table 1). To investigate the transcriptional corepressor activity of BCOR, we focused on a gene-specific transcriptional regulatory protein and found that nucleus factor 1B (*NFIB*) and distal-less homeobox 5 (*DLX5*), and ZFPM2 were the most significantly upregulated genes with a ratio of more than 15 (Table 2). Consistent with these results, qRT-PCR showed that these genes were significantly expressed in Mut PDL cells (Figure 1A).

**TABLE 1:** Upregulated protein class/gene in PDL cells from the OFCD patient compared to a healthy subject.

PANTHER protein class/gene	*Homo sapiens* reference list	Expected	Fold enrichment	*p*-value
Extracellular matrix protein	166	1.06	4.72	4.71E-03
Chromatin/chromatin-binding, or -regulatory protein	209	1.33	3	4.68E-02
Cytoskeletal protein	510	3.25	2.15	4.67E-02
Cell adhesion molecule	90	0.57	5.23	2.14E-02
Ion channel	146	0.93	4.3	1.54E-02
Gene-specific transcriptional regulatory protein	1096	6.99	2	1.68E-02
Unclassified	11455	24.72	1.01	0.00E+00

According to the PANTHER database system, various signaling pathways were significantly implicated in upregulated genes with log2 ratios > 4, which included 155 genes.

**TABLE 2:** Genes involved in the gene-specific transcriptional regulatory protein group.

PANTHER protein class/gene	Ratio
*NFIB* ^a^	19.93
*DLX5* ^a^	16.49
*ZFPM2* ^a^	15.48
*ZIC3*	7.01
*TSHZ2*	6.55
*BARX1*	6.15
*IRX1*	5.72
*ID4*	5.53
*IRX5*	5.31
*ZFHX4*	4.84
*MECOM*	4.26
*DACH1*	4.11

a Genes were the most significantly upregulated genes in the gene-specific transcriptional regulatory protein group, with a ratio of more than 15.

To determine how BCOR influences cells during hyperactive root development, we first overexpressed WT BCOR in the HPdLF cells. Immunofluorescence assay results showed that WT BCOR was also expressed in the nuclei of HPdLF cells after transfection (Figure 1B). The proliferation of apical papillary cells during root development may be a key element in the etiology of radiculomegaly. According to our previous study, BCOR Mut PDL cells grow faster than healthy cells (Surapornsawasd et al., 2014). WT BCOR-transfected HPdLF cells showed a significant reduction in cell number after overexpression (Figure 1C). After WT BCOR overexpression in HPdLF cells, the expression of *NFIB*, *DLX5*, and ZFPM2 was verified using qRT-PCR (Figure 1D). The expression of *DLX5* was upregulated, that of *NFIB* remained unchanged, and that of ZFPM2 was downregulated. As a transcriptional corepressor, BCOR inhibits the expression of its downstream genes (Huynh et al., 2000). Therefore, we focused on ZFPM2 as a direct regulator of BCOR.

### Identifying the interacting corepressor downstream genes

**FIGURE 2:**
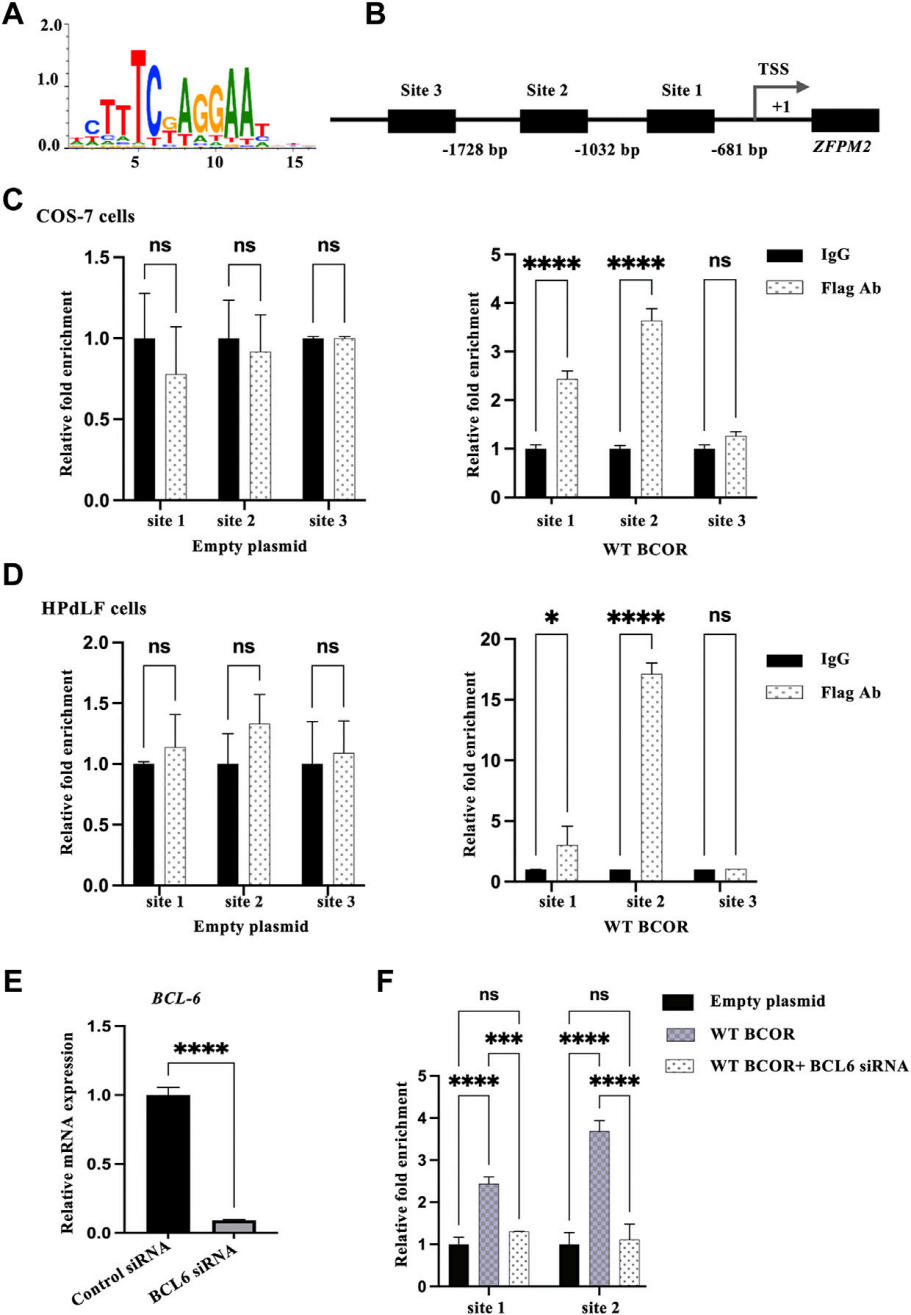
Identifying BCOR downstream genes. **(A)** BCL6 consensus logo. **(B)** Prediction of BCL6 binding sites in the ZFPM2 promoter region. Three BCL6 binding sites within 2 kb of exon 1 of ZFPM2 were identified using LASAGNA-Search 2.0. TSS represents transcription start site. **(C,D)** ChIP assay for the occupation of BCL6 on the promoter of ZFPM2 in COS-7 and HPdLF cells. IgG, *n* = 3; empty plasmid/WT BCOR, *n* = 3. Data are expressed as the means ± standard deviations from triplicate experiments. ANOVA was used to assess differences in fold change within each group. *p < 0.05, ****p < 0.0001; ns, not significant. **(E)** Validation of BCL6 suppression in COS-7 cells using qRT-PCR. *t*-test, ****p < 0.0001. **(F)** BCL6 knockdown co-transfected with WT BCOR failed to recruit promoter binding sites in COS-7 cells. Data are expressed as the means ± standard deviations from triplicate experiments. ANOVA, ****p < 0.0001; ns, not significant.

The ability of transcription factors to recognize and dynamically bind to degenerate sequence motifs in promoters is critical for transcription (Wong et al., 2015), whereas the BCOR/BCL6 complex represses downstream genes (Ghetu et al., 2008). Thus, we hypothesized that the BCOR/BCL6 complex might directly regulate the expression of ZFPM2, which was considerably overexpressed in the OFCD patient and downregulated after WT BCOR overexpression in HPdLF cells; we first tried to find the underlying processes for the elevation of genes related to BCOR-Mut OFCD patients. To test this hypothesis, we performed a ChIP assay using primers spanning the putative BCL6 responsive element region within the 2 kb flanking region of the ZFPM2 gene. These sequences, which included 2 kb upstream of each transcription start site, were examined for transcription-binding sites defined in LASAGNA-Search 2.0. Binding site enrichment was determined using a *p*-value of < 0.001, which was considered significant (Figure 2A). Within 2 kb of the ZFPM2 gene, three potential transcription factor-binding sites were identified (Figure 2B). The ChIP assay revealed that WT BCOR/BCL6 could bind to promoter sites 1 and 2 in COS-7 cells and HPdLF cells (Figures 2C,D). Following BCL6 knockdown in COS-7 cells (Figure 2E), WT BCOR could not be recruited to the promoter regions (Figure 2F). Our findings suggest that WT BCOR binds to the promoter region of ZFPM2 *via* BCL6.

### Mut interacting corepressor failed to regulate zinc finger protein multitype 2 expression

**FIGURE 3:**
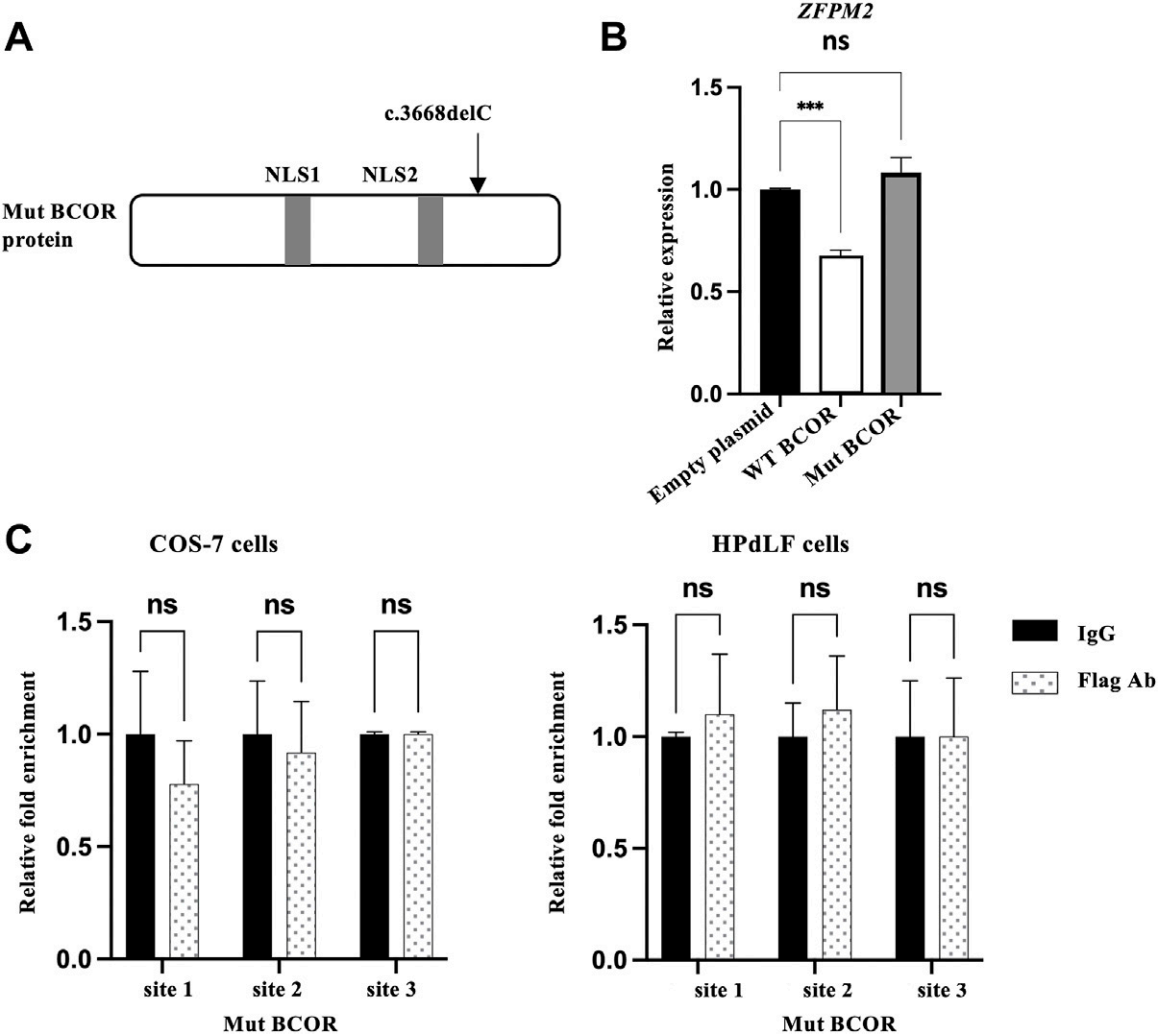
Mut BCOR failed to bind the BCL6 promoter sites on ZFPM2. **(A)** The Mut BCOR plasmid (c.3668delC) have a nuclear localization signal (NLS1 and NLS2), which is the same genotype as that of our patient from our previous research. **(B)** Expression of ZFPM2 mRNA after WT or Mut BCOR overexpression in HPdLF cells. WT BCOR repressed ZFPM2 expression, but Mut BCOR did not. *t*-test, ***p < 0.001; ns, not significant. **(C)** ChIP assay for the occupation of BCL6 on the promoter of ZFPM2 in Mut BCOR-overexpressed COS-7 cells and HPdLF cells. Mut BCOR failed to bind BCL6 binding sites 1, 2, and 3 in the ZFPM2 promoter region in COS-7 and HPdLF cells. ANOVA, ns = not significant.

The Mut BCOR protein (Figure 3A), the same genotype as of our patient that carried two nuclear localization signals (NLS1 and NLS2), was expressed in the nucleus as reported by us previously (Surapornsawasd et al., 2015). To determine whether Mut BCOR controls ZFPM2 expression, we overexpressed the Mut BCOR plasmid in HPdLF cells to investigate its function; however, ZFPM2 expression remained unchanged (Figure 3B). Next, we checked the binding of Mut BCOR to the same ZFPM2 promoter regions using the ChIP assay. After immunoprecipitation, Mut BCOR overexpression failed to recruit any of these binding sites in COS-7 and HPdLF cells (Figure 3C). Our findings suggest that BCOR fails to regulate ZFPM2 expression *via* BCL6 in OFCD patients with c.3668delC.

### Zinc finger protein multitype 2 regulates alkaline phosphatase expression in osterix and miRNA in the tooth development pathway

**FIGURE 4:**
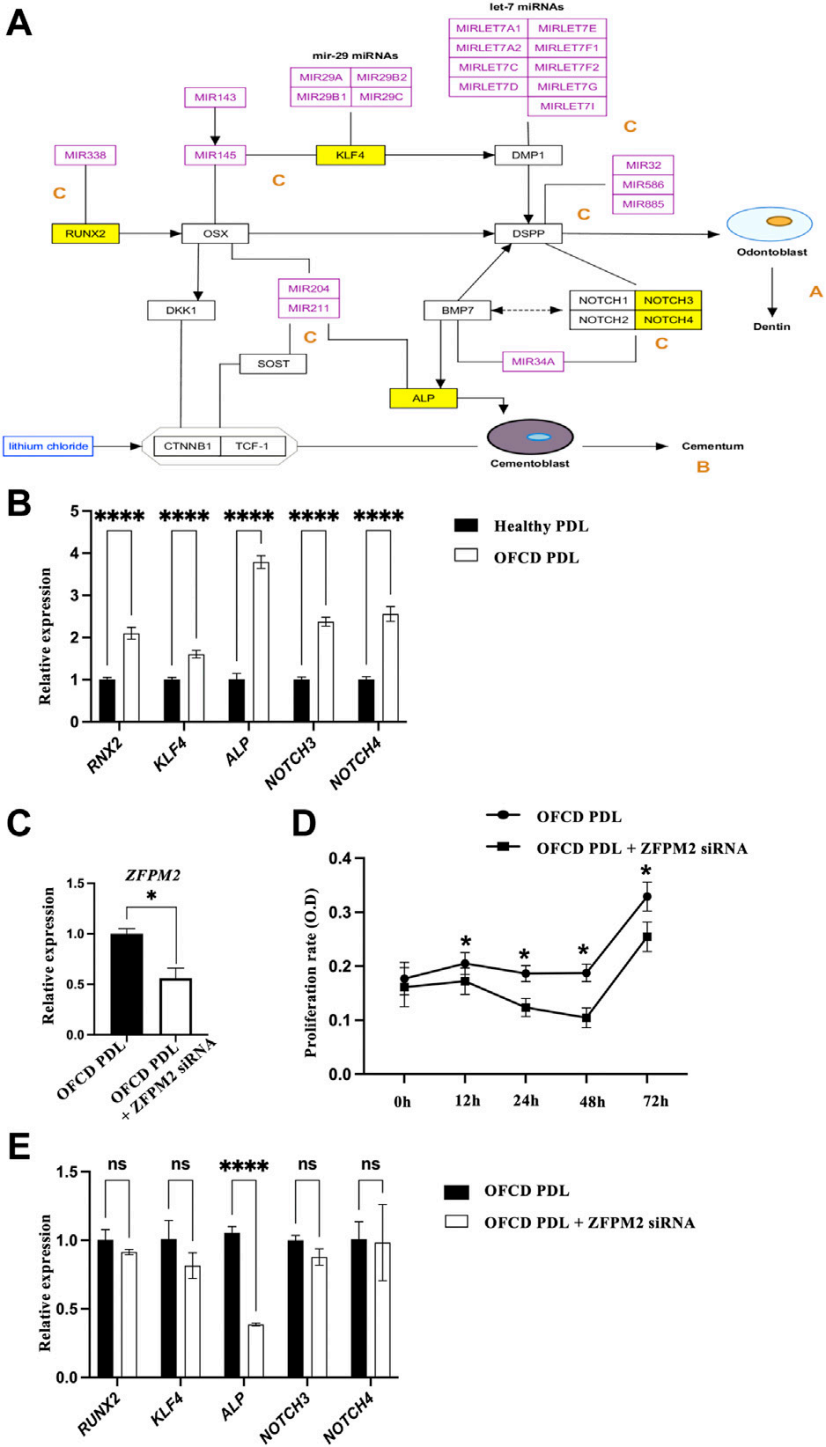
ZFPM2 regulates ALP expression in Osx and miRNA in the tooth development pathway. **(A)** Osx and miRNA in the tooth development pathway. Compared with that in the healthy subject, *RUNX2*, *KLF4*, *NOTCH3*, *NOTCH4*, and ALP were considerably elevated in the patient with the OFCD syndrome. A) Osx control of Dspp demonstrates its significance in dentinogenesis. B) Osx controls cementoblast differentiation via the Wnt-β-catenin pathway. C) Role of miRNAs in fine-tuning tooth development. The yellow boxes indicate the elevated signals above a fold change of 2 in the microarray analysis. **(B)** The expression of differentially expressed genes was determined using qRT-PCR. ANOVA, ****p < 0.0001. **(C)** qRT-PCR validation of ZFPM2 suppression in Mut PDL cells. *t*-test, *p < 0.05. **(D)** MTT assays showed that Mut PDL cell proliferation was inhibited after ZFPM2 knockdown. Points and bars indicate the means and standard deviations of the triplicate values. ANOVA, *p < 0.05. **(E)** After ZFPM2 inhibition, mRNA ALP expression was significantly reduced in Mut PDL cells whereas the mRNA expression of *RUNX2*, *KLF4*, *NOTCH3*, and *NOTCH4* did not change. ANOVA, ****p < 0.0001.

GO pathway analysis revealed that 66 pathways were upregulated in Mut PDL cells. Prostaglandin synthesis and regulation, the role of expression in osterix (Osx) and miRNAs in tooth development, the serum response factor and miRNA pathway, primary focal segmental glomerulosclerosis, and the neural crest differentiation pathway were the most significant upregulated pathways (Table 3). The role of Osx and miRNAs in tooth development was most closely associated with dental root development, in which runt-related transcription factor 2 (*RUNX2*), Krüppel-like factor 4 (*KLF4*), notch receptor 3 (*NOTCH3*), notch receptor 4 (*NOTCH4*), and alkaline phosphatase (ALP) expression was considerably elevated in the OFCD patient, with fold changes of 2.22, 2.10, 2.25, 2.07, and 2.62, respectively (Figure 4A). The expression of these genes was validated using qRT-PCR (Figure 4B). Next, we investigated whether ZFPM2 regulates any of these signaling pathways in radiculomegaly. Cell proliferation was hampered when ZFPM2 was silenced in OFCD PDL cells (Figures 4C,D). After ZFPM2 inhibition in the OFCD PDL cells, the expression of ALP was significantly reduced, whereas the expression of *RUNX2*, *KLF4*, *NOTCH3*, and *NOTCH4* was unchanged (Figure 4E). These findings imply that the BCOR corepressor mutation failed to control the expression of *RUNX2*, *KLF4*, *NOTCH3*, *NOTCH4*, and ALP in Osx and of miRNAs in the tooth development pathway. ALP is an early important marker during the differentiation of osteoblasts, odontoblasts, and cementoblasts (Basdra and Komposch, 1997; Komaki et al., 2012; Kadokura et al., 2019). In patients with OFCD syndrome, BCOR regulates ZFPM2, which in turn regulates ALP expression during tooth root elongation.

**TABLE 3:** Pathway enrichment in OFCD syndrome.

Pathway	*p*-value	Matched entities	Pathway entities of experiment type
Prostaglandin synthesis and regulation	2.65E-06	10	46
Role of Osx and miRNAs in tooth development	1.28E-04	5	37
SRF and miRNAs in smooth muscle differentiation and proliferation	2.86E-04	4	17
Neural crest differentiation	6.89E-04	11	101
Primary focal segmental glomerulosclerosis	7.56E-04	9	74
Cell-type dependent selectivity of CCK2R signaling	8.94E-04	4	13

In GO and pathway enrichment analysis, upregulated genes with log2 ratios > 4 are shown.

## Discussion

BCOR plays an essential role in human development as a transcriptional corepressor, and the activity of BCOR during embryonic development has also been investigated in an animal model. The partial inhibition of bcor causes developmental abnormalities in the eye, skeleton, and central nervous system in zebrafish, partially mimicking the clinical appearance of human patients with OFCD syndrome (Ng et al., 2004). Hamline et al. created a conditional exon-deletion *Bcor*-allele mouse to investigate the roles of BCOR and, by extension, the polycomb repressive complex 1.1 (PRC1.1). The *Bcor* mutation was lethal in male embryos at early embryonic ages and females that were heterozygous, exhibiting mosaic expression due to the X-linkage of the gene, had lower postnatal viability and OFCD-like abnormalities; however, they did not mention radiculomegaly in the *Bcor* Mut mice (Hamline et al., 2020). Radiculomegaly in OFCD patients is mainly seen on permanent incisors, canines, and premolars. In humans, two renewals of the dentition occur through life, corresponding to diphyodonty. Monophyodont teeth in rodents refer to single tooth generation in which the molars have limited growth whereas incisors are continuously growing (Whitlock and Richman, 2013). As a result, the Bcor knockout mice failed to show radiculomegaly because of the difference in dentition pattern. Previously, only a few confirmed cases of nonsyndromic/nonfamilial radiculomegaly had been reported but the underlying molecular mechanism remains unclear (Smith et al., 2018). In our study, we analyzed the affected tissue of an OFCD patient to elucidate the genetic regulatory mechanism and the influences on phenotype.

A total RNA microarray revealed alterations in the expression of numerous genes in BCOR Mut PDL cells, several of which are implicated in transcriptional upregulation; *NFIB*, *DLX5*, and ZFPM2 were the most significantly upregulated genes in OFCD syndrome. As a transcriptional corepressor, BCOR suppresses the expression of its downstream genes in the nucleus. *DLX5* expression was upregulated, *NFIB* expression was unaltered, and ZFPM2 expression was downregulated after BCOR overexpression in HPdLF cells. Therefore, we assumed that BCOR may influence ZFPM2 directly, whereas the expression of *DLX5* and *NFIB* is regulated indirectly.

After immunoprecipitation of WT BCOR-overexpressing COS-7 cells and HPdLF cells with flagged antibodies, the ChIP assay revealed that WT-BCOR/BCL6 could bind to promoter sites 1 and 2; however, Mut BCOR-overexpressing cells failed to recruit any of these binding sites. We performed BCL6 siRNA and WT BCOR overexpression in COS-7 cells to determine whether BCOR could bind to the promoter regions with or without BCL6. Following BCL6 knockdown, WT BCOR could not be recruited to the promoter regions. Our results showed that BCOR regulates ZFPM2 *via* BCL6 as its transcriptional corepressor, consistent with the finding that BCOR interacts with a subgroup of proteins within the POZ domain, including the nucleoplasmic POZ/zinc finger transcription repressor BCL6 (Huynh et al., 2000). BCOR has several interacting regions in addition to BCL6, including AF9 (a region that interacts with AF9, the common mixed-lineage leukemia fusion partner), NSPC1 (a region that interacts with nervous system polycomb1), and Ankyrin (a region involved in protein–protein interaction) (Ghetu et al., 2008). The c.3668delC Mut BCOR protein (p.S12223Wfs*15) has a molecular size of 134.18 kDa and affects the AF9 region and lacks the Ankyrin region, whereas the WT BCOR has a molecular size of 188.23 kDa, as confirmed by western blot analysis in our previous study (Surapornsawasd et al., 2015). In this study, Mut BCOR failed to repress ZFPM2, the BCOR repressive target whereas WT BCOR did repress it. Mut BCOR also failed to recruit BCL6 binding sites in the promoter region of ZFPM2 although it was expressed in the nucleus. The data suggested that the truncated protein apparently lacks BCL6-binding function in the C terminal without AF9 and the Ankyrin region; however, further research is needed in the future.

ZFPM2 regulates the activity of GATA binding protein 4 (GATA4), which is a key regulator that can both activate and downregulate its downstream genes. ZFPM2/FOG family member 2 (FOG2) has been identified as a key regulator of many fundamental biological processes (Tevosian et al., 2000; Fossett et al., 2001; Hirai et al., 2004). However, little is known about its role in tooth development. GATA4 has been identified as a novel regulator of root development and is important for odontoblast polarity because it promotes the growth and differentiation of dental mesenchymal cells by regulating glucose metabolism (Zhang et al., 2020). Further study is needed to determine whether ZFPM2 and its cofactor GATA4 interact with BCOR during odontogenesis. ZFPM2 and its cofactor GATA4 are also expressed in the human heart during development and regulate genes involved in cardiac morphogenesis. In FOG2 knockout mouse embryos, a thin ventricular myocardium, common atrioventricular canal, tetralogy of Fallot malformation including ventricular septal defect, pulmonary stenosis, misplaced aorta, and an absence of coronary vasculature have all been reported (Tevosian et al., 2000; Hirai et al., 2004). Interestingly, 67% of OFCD patients whose cardiac function has been examined had at least one cardiovascular defect in which septal defects, such as atrial, ventricular, or both, were present. Other cardiovascular anomalies include patent ductus arteriosus (6%) and a double outlet right ventricle (3%) (Ragge et al., 2019; Hamline et al., 2020). The BCOR mutation resulted in overexpression of the transcription factor ZFPM2, which could be linked to cardiac stem cell development.

Bcor is expressed in both the dental epithelium and the mesenchyme during the early stages of tooth development. BCOR mutations are linked to dental anomalies, most of which are “deficiency defects,” such as oligodontia/hypodontia. BCOR in mesenchyme performs crucial roles in cellular events, such as apoptosis and cellular differentiation (Cai et al., 2010). The formation of tooth root commences after crown development. Therefore, radiculomegaly and hyperdontia appear to be diametrically opposed. Uncontrollable root growth appears to be caused by a few factors in patients with OFCD syndrome (Kantaputra, 2014). Organ sizes in *Homo sapiens* must be controlled by mechanisms, such as the closure of anterior and posterior neuropores (Nikolopoulou et al., 2017). In patients with OFCD syndrome, this mechanism appears to be dysfunctional in the control of root growth. By disruption of the epigenetic mechanism and reactivation of transcription of target genes, BCOR mutation led to the upregulation of AP2α, the repressive target of BCOR, resulting in increased osteo–dentinogenic potential of mesenchymal stem cells (MSCs) derived from the apical papilla of an affected patient with a frameshift mutation in c.2613delC (Fan et al., 2009). Consistent with our results, BCOR mutations result in abnormal reactivation of several molecules, including ZFPM2, a downstream target of BCOR, resulting in increased cemento–dentinogenic potential of PDL cells.

We show that the roles of Osx and miRNAs in tooth developmental pathways are highly important, with RUNX2, KLF4, NOTCH3, NOTCH4, and ALP being considerably elevated in our OFCD patient. These findings show that mutant PDL cells have higher levels of dentinogenetic and cementogenetic regulatory factors, which could have led to radiculomegaly. ZFPM2 inhibition in the PDL cells of the OFCD patient showed that the expression of ALP was significantly reduced, whereas the expression of *RUNX2*, *KLF4*, *NOTCH3*, and *NOTCH4* was unchanged. Furthermore, the suppression of ZFPM2 also inhibited cell proliferation. Our findings suggest that BCOR failed to repress its downstream genes, resulting in elevated ZFPM2 and ALP expression and subsequent upregulation of cell proliferation, ultimately leading to hyperactive root formation in OFCD syndrome. ALP is an important early differentiation marker for osteoblasts, odontoblasts, and cementoblasts (Basdra and Komposch 1997; Komaki et al., 2012; Kadokura et al., 2019). The proliferation of HERS cells, which later fragment into the PDL and cementum, is required for root-end closure during root growth. PDL is a multifunctional connective tissue unit that includes osteoblasts, osteoclasts, fibroblasts, ERM, odontoblasts, cementoblasts, macrophages, and undifferentiated mesenchymal cells (Zeichner-David et al., 2003; Rincon et al., 2006; Tsunematsu et al., 2016). In our study, the PDL cells from the apical region of the affected tissue contained a variety of cells. Consequently, further research is needed to determine which cells are affected by the BCOR mutation during tooth development.

The NMD mechanism has the potential to degrade mutant mRNA with a premature stop codon, resulting in significantly lower mRNA levels and translation (Frischmeyer and Dietz, 1999). According to a previous study, the PTC-induced NMD process leads to unstable mutant transcripts and increased cell proliferation in PDL cells, indicating that mutant cells are most likely haploinsufficient for the WT BCOR protein (Surapornsawasd et al., 2014). Consistently, mutant cells have an insufficient level of the corepressor WT BCOR protein, resulting in the upregulation of several molecules. When one copy of a gene is deleted or contains a loss-of-function mutation, the normal dosage product generated by the single wild-type gene is insufficient for complete function. Diseases caused by haploinsufficiency are usually due to mutations in genes encoding proteins that are required in large amounts or in genes encoding regulatory molecules whose concentrations are closely titrated within the organism. Most BCOR mutation types (insertion/deletion/frameshift) undergo the NMD mechanism, which results in haploinsufficiency. Therefore, all BCOR gene mutations result in similar phenotypes and severity variations, but interestingly, almost all affected individuals have a unique feature, that is, radiculomegaly (Surapornsawasd et al., 2015). The genotype–phenotype correlation is extremely complex and cannot be explained solely by the mutation site. Other factors, such as mosaicism from X inactivation patterns and individual sensitivities to protective mechanisms, may play a role in the severity of this syndrome. Using the affected tissue of a diseased individual with c.3668delC frameshift mutation would help understand the basic pathogenesis of radiculomegaly resulting from WT BCOR haploinsufficiency in all affected patients.

When investigating rare diseases, small sample sizes are inevitable, limiting research progress on the complete understanding of the pathogenesis of radiculomegaly and cardiovascular anomalies. Nonetheless, ZFPM2 has been identified as a possible BCOR downstream gene that may play a role in radiculomegaly pathogenesis. The detailed mechanism by which ZFPM2 is involved in hyperactive root formation needs to be clarified in future research. Our findings imply that the BCOR mutation alone does not account for the phenotypic abnormalities of the disease. The study of patients with rare hereditary diseases, such as the OFCD syndrome, has led to the discovery of a molecular pathway that could explain aberrant tooth root formation, cardiovascular disorders, and the origin of the phenotype in another organ.

## Conclusion

In this study, we show that a BCOR mutation induced ZFPM2 regulation *via* BCL6, subsequently regulating ALP expression and cellular proliferation and possibly contributing to hyperactive root formation in the OFCD syndrome. Our findings provide an important reference for rare genetic diseases.

## Data Availability

The datasets presented in this study can be found in online repositories. The names of the repository/repositories and accession number(s) can be found below: https://www.ncbi.nlm.nih.gov/geo/, GSE19870.
