# Supplementary material for: Molecular mechanism of hyperactive tooth root formation in oculo-facio-cardio-dental syndrome
Source: Front Physiol. 2022 Jul 25;13:946282. doi: 10.3389/fphys.2022.946282 (PMC9359619; doi:10.3389/fphys.2022.946282)
Supplement: Supplementary file 1 [file Table1.DOCX]

**Supplementary data**

Primers used for qRT-PCR validation.

| Genes | Forward primers | Reverse primers |
| --- | --- | --- |
| DLX5 | CAACTTTGCCCGAGTCTTCA | GTTGAGAGCTTTGCCATAGG |
| ZFPM2 | TCTGAGGGTGAAGAGCTAATTG | CATCCCTTCTGTGAGAGTCATC |
| NFIB | CACATTGCACAAACCCAGCA | CTTCCTGATTGTCCAGAATC |
| ALP | CCAAAGGCTTCTTCTTGCTG | CCACCAAATGTGAAGACGTG |
| BCL6 | TGTTGATGCTTTCGTCTCCA | CCCCAGCTATGATTTGCACT |
| RUNX2 | ATGATGACACTGCCACCTCTGA | GGCTGGATAGTGCATTCGTG |
| NOTCH3 | TCTCAGACTGGTCCGAATCCAC | ACACTTGCCTCTTGGGGGTAAC |
| NOTCH4 | ATGCGAGGAAGATACGGAGTGG | TCGGAATGTTGGAGGCAGAAC |
| KLF4 | CAGGTGCCCCAGCTGCTTCG | CCCGCCAGCGGTTATTCGGG |
| GAPDH | GACAGTCAGCCGCATCTTC | GCGCCCAATACGACCAAAT |

Primers used in ChIP assay

| BCL6 binding sites | Forward primers | Reverse primers |
| --- | --- | --- |
| Site I | GGCAAGGTGGACTACA | AAAGGAGGAGCGCG |
| Site II | GGCAAGTGCGGAC | CTTGGCAGGAAGGTTA |
| Site III | CCCCTTTTCTCTCTGA | TCGGAGATGAGCAAGC |
